# Effect of Milling Processing Parameters on the Surface Roughness and Tool Cutting Forces of T2 Pure Copper

**DOI:** 10.3390/mi14010224

**Published:** 2023-01-15

**Authors:** Fuqiang Lai, Anqiong Hu, Kun Mao, Zhangbin Wu, Youxi Lin

**Affiliations:** School of Mechanical Engineering and Automation, Fuzhou University, Fuzhou 350116, China

**Keywords:** T2 pure copper, surface roughness, cutting force, tool displacement acceleration, machining parameters optimization

## Abstract

In this paper, the responses of machined surface roughness and milling tool cutting forces under the different milling processing parameters (cutting speed *v*, feed rate *f*, and axial cutting depth *a_p_*) are experimentally investigated to meet the increasing requirements for the mechanical machining of T2 pure copper. The effects of different milling processing parameters on cutting force and tool displacement acceleration are studied based on orthogonal and single-factor milling experiments. The three-dimensional morphologies of the workpieces are observed, and a white-light topography instrument measures the surface roughness. The results show that the degree of influence on *S_a_* (surface arithmetic mean deviation) and *S_q_* (surface root mean square deviation) from high to low level is the *v*, the *f*, and the *a_p_*. When *v* = 600 m/min, *a_p_* = 0.5 mm, *f* = 0.1 mm/r, *S_a_* and *S_q_* are 1.80 μm and 2.25 μm, respectively. The cutting forces in the three directions negatively correlate with increased cutting speed; when *v* = 600 m/min, *F_x_* reaches its lowest value. In contrast, an increase in the feed rate and the axial cutting depth significantly increases *F_x_*. The tool displacement acceleration amplitudes demonstrate a positive relationship. Variation of the tool displacement acceleration states leads to the different microstructure of the machined surfaces. Therefore, selecting the appropriate milling processing parameters has a positive effect on reducing the tool displacement acceleration, improving the machined surface quality of T2 pure copper, and extending the tool’s life. The optimal milling processing parameters in this paper are the *v* = 600 m/min, *a_p_* = 0.5 mm, and *f* = 0.1 mm/r.

## 1. Introduction

Pure copper has excellent electrical and thermal conductivity, high elasticity, high ductility, and good corrosion resistance. Hence, it has been widely used in automobile industry manufacturing, electronic communication, aerospace, and many other fields [1,2,3]. Higher demands are placed on the cutting process and the machining quality of pure copper material in the actual machining process. Complex cutting techniques and high material plasticity often lead to the adhesion of workpiece materials, displacement acceleration of tools, plastic deformation of machined surfaces, and severe tool wear problems, reducing the cutting quality and processability of pure copper material [4]. Therefore, to meet the industry’s growing demands and improve the machining quality of the pure copper part, it is particularly important to study the cutting process and surface quality of pure copper material.

Regular and standard machined surface topography is the primary prerequisite to ensuring the service performance of parts [5]. Therefore, to improve the cutting quality of the pure copper part, it is necessary to study the formation process of the machined surface topography during cutting. Many researchers use surface roughness to evaluate surface topography and to study the variation trend of surface topography under different processing parameters through cutting tests [6,7]. Su et al. [8] used the orthogonal test method to analyze the roughness variation trend of the machined surface at different cutting speeds. They investigated the basic method to obtain optimized surface quality. The experimental results showed a close relationship between the roughness of the machined surface and the chip formation process. Yue et al. [9] summarized the influence of processing parameters on the morphology of the machined surface from the perspective of material properties. The authors concluded that although each process parameter has similar influence rules for different machined materials, the degree of influence is different. Paturi et al. [10] established a surface roughness model using a multi-objective optimization method and analyzed the influence of different processing parameters on surface roughness. The feed rate had the strongest influence, followed by cutting speed and depth.

In the actual cutting process, the machining path of the tool is often affected by the cutting speed, feed rate, and other processing parameters. Hence, the displacement acceleration state of the tool constantly varies, affecting the surface morphology of the workpiece [11,12]. Zhang et al. [13] proposed a method to control the integrity of milled surfaces to explore the effect of cutting vibration on the topography of the machined surface. With an increase in the cutting speed and spindle vibration amplitude, the hardening effect of the workpiece was gradually weakened, and the surface topography of the workpiece was optimized. Babu et al. [14] used the response surface method to analyze tool vibration and surface roughness. Moreover, the authors adopted multi-response optimization technology to reduce the cutting surface roughness and tool displacement acceleration amplitude as the optimization objective while determining the optimal cutting parameters. In addition, Kumar et al. [15] optimized the cutting speed, feed rate, and cutting depth of magnesium alloy milling via multi-response optimization technology. Lastly, the authors evaluated and recommended optimal milling process parameters by using analysis of variance (ANOVA).

**Figure 1:**
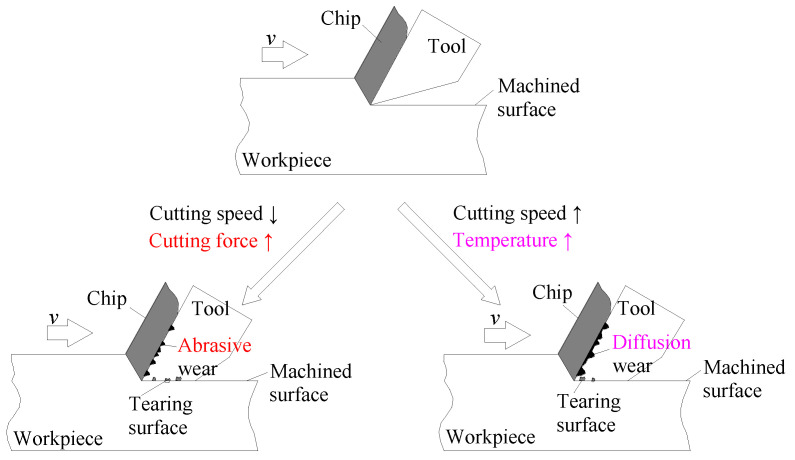
The schematic diagram of tool wear and surface defects in pure copper milling.

In the manufacturing industry, the service performance of mechanical parts is closely related to their surface quality [16,17]. As shown in Figure 1, the metallic material with high plasticity, such as copper and aluminum alloys, are influenced by the high temperature and high contact pressure in the local cutting deformation area. The workpiece materials easily adhere to the surface of the tools, forming the phenomenon of tool sticking. This phenomenon causes displacement acceleration of the tool and aggravates tool wear, including abrasive and diffusion wear. Tool sticking phenomenon would also damage the machined surfaces. Therefore, studying the internal relationships between machining processing parameters, tool displacement acceleration and machined surface quality is very important.

**Figure 2:**
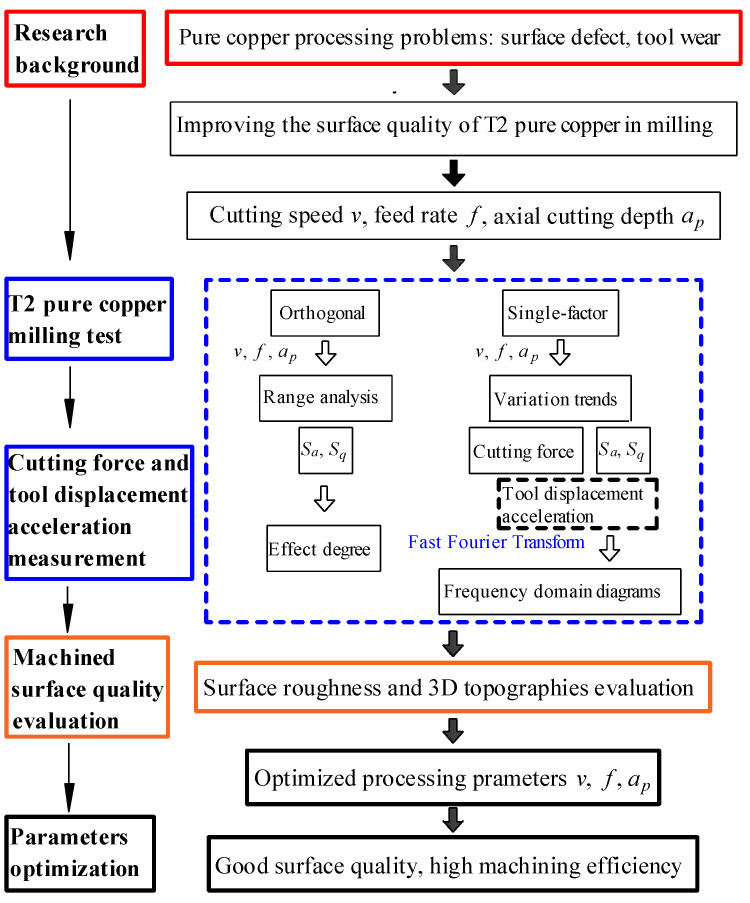
The framework of the study and research technology roadmap.

The goal of this study is to improve the processed surface quality of T2 pure copper. This article provides evaluation and measurement methods for the surface topography of pure copper, and the surface roughness evaluation method is expressed in Section 2.2 by using the surface arithmetic mean deviation *S_a_*, the surface root mean square deviation *S_q_* and the range analysis. Based on the results of orthogonal and single-factor tests, the degree to which cutting speed *v*, feed rate *f*, and axial cutting depth *a_p_* affect the surface roughness of the machined surface are characterized in Section 3.1. According to the results of the single-factor test, the influence rules of *v*, *f*, *a_p_* on the cutting force of the tool during milling are shown in Section 3.2. In Section 3.3, the influence rules of *v*, *f*, *a_p_* on the tool displacement acceleration during milling are shown, the frequency domain diagrams are obtained by the Fast Fourier Transform, and the relationship between the tool displacement acceleration state and the surface morphology is then studied. The conclusions of this article are presented in Section 4. The research results of this paper can provide a theoretical basis and technical guidance for high-quality and high-efficiency milling of T2 pure copper. Figure 2 illustrates the framework of the study and research technology roadmap.

## 2. Experimental Details

### 2.1. Experimental Setups

T2 pure copper, a non-ferrous metal with good mechanical properties, an ultimate tensile strength of 348 MPa, and a hardness of 105 HV, was selected in this study. Its chemical composition is shown in Table 1. The workpiece size is 200 mm × 100 mm × 10 mm.

**Figure 3:**
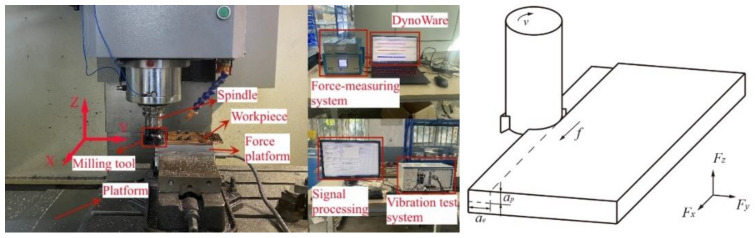
Milling test equipment and schematic diagram of milling processing.

The milling equipment used in the series experiments is a vertical machining center (VMC-850E, Shenyang No.1 machine tool works, Shenyang, China) with a maximum spindle speed of 8000 rpm. C-W-Co cemented carbide was chosen as the material of the milling cutter (APKT1604PDER-MA, Boen, Taizhou, China), the cutter corner radius was 0.2 mm, the main cutting edges angle was 90°, the rake angle was 8°, and the flank angle was 11°. The milling cutter was installed on the milling cutter plate with a diameter of 63 mm, and four milling blades can be installed. The cutting force was collected by a dynamometer (9257B, Kistler, Zurich, Switzerland), and software (DynoWare, Kistler, Zurich, Switzerland) was used for the force signal real-time monitoring and analysis. During the milling machining experiments, a data recorder (NI PXIe-1078, NI, Austin, TX, USA) with a sound and vibration module (NI PXIe-4492 PXI, NI, Austin, TX, USA) was used to collect the displacement acceleration signal of machine spindle, and the sampling frequency was set to 2000 Hz. Three-dimensional morphologies of the workpieces were observed using a white-light topography instrument (Micromesure 2, STIL, Aix-en-Provence, France), and the surface roughness was measured by a matched software (Gwyddion, Open source). The cutting force and tool displacement acceleration tests were performed using a single-factor method, and the specific test parameters are listed in Table 2. The milling test equipment and schematic diagram of the process are shown in Figure 3.

### 2.2. Evaluation and Measurement of Surface Morphologies of Pure Copper

**Figure 4:**
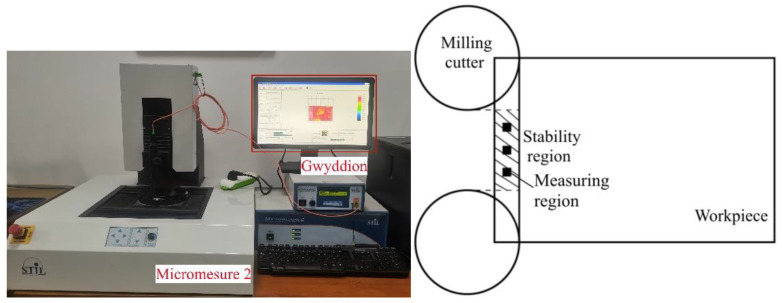
Surface roughness measurement equipment and method.

In this study, the two positions where the milling cutter head radius is completely cut into and cut out of the workpiece surface are selected as the start and end positions of the stable milling area. The surface roughness is measured and averaged in three 0.5 mm × 0.5 mm areas with the same interval in the middle of the stable milling area to ensure the accuracy and authenticity of the test. The machined surface’s three-dimensional (3D) signal is collected by a white-light topography instrument. The surface signal is decomposed by Gwyddion software to characterize and evaluate the morphology of the processed surface. The measurement method and equipment are shown in Figure 4.

**Figure 5:**
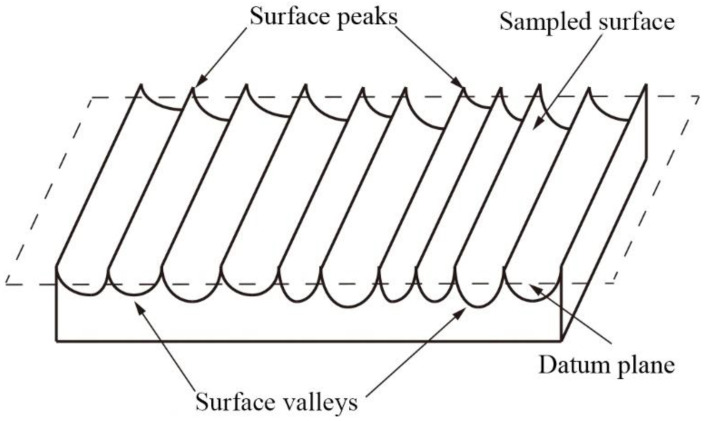
Schematic diagram of 3D surface roughness of body surface.

Three-dimensional roughness means adding a dimension based on the two-dimensional roughness to evaluate and characterize the 3D morphologies of the machined surfaces. The principle is to establish the datum plane of the sampling area by the least squares method and to calculate the surface roughness parameters based on the height of the surface profile. The schematic diagram of a 3D surface roughness is shown in Figure 5. The evaluation parameters of 3D surface roughness, especially the *S_a_* and the *S_q_*, significantly affect the fatigue life and frictional properties of parts. Many scholars also use the two standards mentioned above to evaluate the surface topography of the workpiece [18,19]. The related expressions of the two parameters are listed in Table 3.

The range analysis method can directly show the influence of each factor of the orthogonal test on the test results and select the optimized combination of factors in the test [20]. The range analysis expression is shown in Equation (1):

(1) $${R=\max(K}_{i})-{\min(K}_{i})$$

where the corresponding indexes of main effect *K_i_* are the sum of test results at *i* (*i* = 1, 2, 3) under each factor. The range *R* is the maximum difference of the corresponding indexes of the main effect under each factor.

## 3. Results and Discussion

### 3.1. The Effect of Milling Processing Parameters on Surface Roughness

#### 3.1.1. Orthogonal Milling Test

The orthogonal milling test is designed preferentially in this study to determine the influence degree of milling processing parameters on the roughness of the machined surfaces and to provide a receivable reference for the subsequent single-factor test. The cutting speed *v*, axial cutting depth *a_p_*, and feed rate *f* are selected as orthogonal factors. Then, an orthogonal milling experiment with three factors and three levels is designed. The specific experiment contents and results are shown in Table 4. A VMC-850E vertical milling machine (Shenyang No.1 machine tool works, Shenyang, China) is used to finish milling T2 pure copper in orthogonal and single-factor tests. After milling, the topography features of the machined surfaces are collected and recorded using a white-light topography instrument (STIL, Aix-en-Provence, France). Lastly, the 3D surface roughness of the workpiece is calculated using Gwyddion software.

Table 5 shows the range of calculation results of *S_a_*. The value of *R* is positively correlated with the ability of the factor to affect the test results. According to Table 5, the range order of each factor is *R_a_* > *R_c_* > *R_b_*, i.e., the influence order of each factor is *v* > *f* > *a_p_*. Therefore, in the milling processing parameters designed in the experiment, the cutting speed *v* has the strongest influence on *S_a_*, followed by the feed rate *f* and the axial cutting depth *a_p_*. In the factor a column, the primary and secondary corresponding indexes are *K*_2_ ˂ *K*_3_ ˂ *K*_1_; in the b factor column, they are *K*_1_ ˂ *K*_2_ ˂ *K*_3_; in the c factor column, they are *K*_1_ ˂ *K*_2_ ˂ *K*_3_. Therefore, the scheme for obtaining the minimum value of *S_a_* is a_2_b_1_c_1_, i.e., the cutting speed is 600 m/min, the milling depth is 0.5 mm, and the feed rate is 0.1 mm/r.

According to Table 6, the influence order list of each factor is *v* > *f* > *a_p_*, i.e., the cutting speed *v* has the strongest influence on *S_q_*, followed by the feed rate *f*, and the axial cutting depth *a_p_*. In the factor column, the primary and secondary corresponding indexes are *K*_2_ ˂ *K*_3_ ˂ *K*_1_; in the b factor column, they are *K*_1_ ˂ *K*_2_ ˂ *K*_3_; in the c factor column, they are *K*_1_ ˂ *K*_2_ ˂ *K*_3_. Therefore, the scheme for obtaining the minimum value of *S_q_* is a_2_b_1_c_1_, i.e., the cutting speed is 600 m/min, the milling depth is 0.5 mm, and the feed rate is 0.1 mm/r.

Based on the above analysis of the range results, the primary and secondary order of each factor in the orthogonal test is the same regardless of *S_a_* or *S_q_*, i.e., *v* > *f* > *a_p_*. Zhang et al. [21] compared the trend of surface roughness under different processing parameters and concluded that higher cutting speed and lower cutting depth can optimize the surface quality of workpiece. During T2 pure copper milling, the cutting speed has the highest influence on the surface roughness of the machined surfaces, followed by the feed rate and the axial cutting depth. The optimal parameters for the surface quality are the cutting speed of 600 m/min, the axial cutting depth of 0.5 mm, and the feed rate of 0.1 mm/r, while the values of *S_a_* and *S_q_* are 1.80 and 2.25 μm, respectively.

#### 3.1.2. Single-Factor Milling Test

Based on the optimal orthogonal test results, the cutting speed *v*, the axial cutting depth *a_p_*, and the feed rate *f* are selected as independent variables of the single-factor test. The influence rules of each processing parameter on the surface morphologies of T2 pure copper are explored by measuring the machined surface roughness under different processing parameters. The parameters of the single-factor milling test are shown in Table 1.

**Figure 6:**
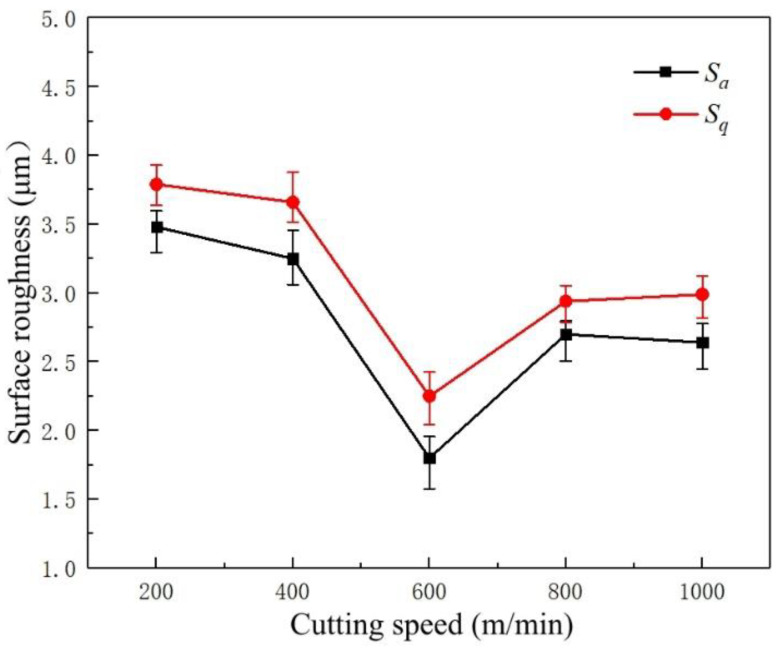
The variation trend of cutting speed on the surface roughness of the machined surfaces.

Figure 6 shows the variation trend of the roughness parameters *S_a_* and *S_q_* of the machined surface at different cutting speeds. The values of *S_a_* and *S_q_* change significantly at different cutting speeds and behave similarly, indicating that the cutting speed is an important factor affecting surface roughness [22]. As the cutting speed increases, the overall surface roughness value of the machined surfaces decreases. When the cutting speed is at lower levels (200 and 400 m/min), the values of *S_a_* and *S_q_* are relatively high, and the surface quality of the workpieces is poor. However, when the cutting speed is high, the surface roughness of the machined surfaces is relatively low. Moreover, the surface quality is improved when the cutting speed is 600 m/min. This is because an increase in the cutting speed reduces the cutting force during cutting, weakening the extrusion friction between the tool and the workpiece, stabilizing the machining process, and reducing the roughness of the machined surface [23].

**Figure 7:**
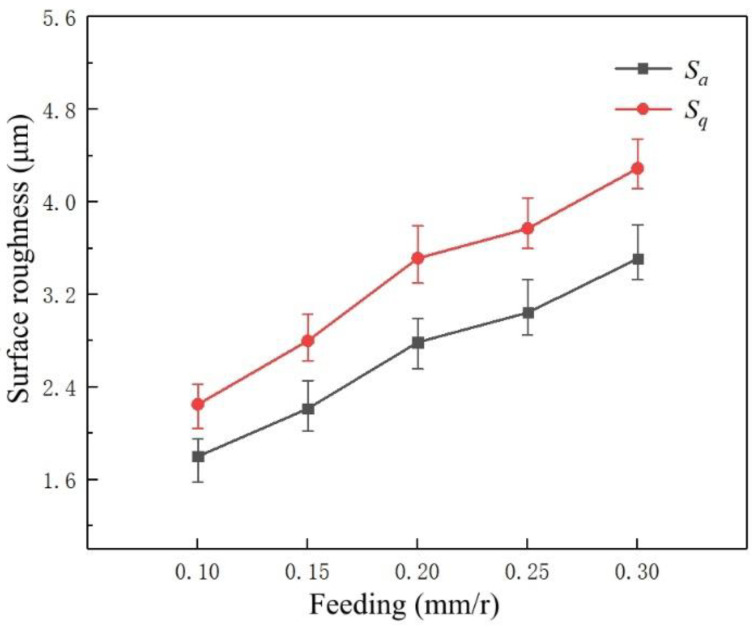
The variation trend of feed rate on the roughness of the machined surfaces.

Figure 7 represents the variation trend of surface roughness parameters *S_a_* and *S_q_* of the machined surfaces at different feed rates. Parameters *S_a_* and *S_q_* demonstrate an upward trend as the feed rate increases. Based on the principle of metal cutting, the relationship between the feed velocity *V_f_*, the feed rate *f_z_* per tooth, and the number of cutter teeth *z* is shown in Equation (2):

(2) $$V_{f}=n\cdot f_{z}\cdot z$$

According to Equation (2), when the cutting speed remains unchanged and the spindle speed remains constant, increasing the feed rate will increase the feed speed per unit of time, increasing the instantaneous cutting area during cutting. As a result, the instantaneous deformation range of the workpiece surface is further increased when milling T2 pure copper. Therefore, the tool must absorb the reaction force of the machined material to resist deformation during milling, gradually increasing the cutting force and affecting the surface quality of the workpieces.

**Figure 8:**
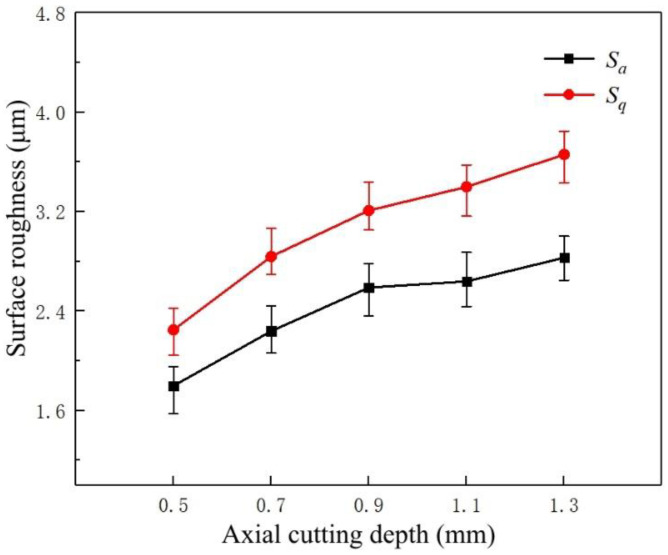
The variation trend of axial cutting depth on the surface roughness of the machined surfaces.

Figure 8 shows the variation trend of surface roughness parameters *S_a_* and *S_q_* at different axial cutting depths. The surface roughness gradually increases with the axial cutting depth. The values of *S_a_* and *S_q_* are significantly increased when the milling depth is increased from 0.5 to 0.9 mm. However, when the milling thickness exceeds 0.9 mm, the surface roughness increases at a relatively low level.

Since the cutting speed and feed rate remained constant during the milling test, a higher cutting depth increased the volume of removed material per unit of time. Simultaneously, an increase in the volume of removed material means that the tool needs to overcome the reaction force generated by the deformation of the pure copper during milling, increasing the instability of the milling process and surface roughness [10]. During the finishing process, when the axial cutting depth is low, the material removal is relatively high due to an increase in the independent variable. Consequently, the surface roughness values of the machined surfaces are significantly increased.

### 3.2. The Effect of Milling Processing Parameters on Cutting Force of Tools

In the previous sections, orthogonal and single-factor milling tests were performed to analyze the degree and regularity of the influence of each process parameter on the surface morphologies of T2 pure copper. To further investigate the formation process of the machined surfaces, the variation trends of cutting force and tool displacement acceleration under different processing parameters are combined in Section 3.2 and Section 3.3 to investigate the relationships between the displacement acceleration states of the tools and the surface topographies during milling.

#### 3.2.1. The Effect of Cutting Speeds

**Figure 9:**
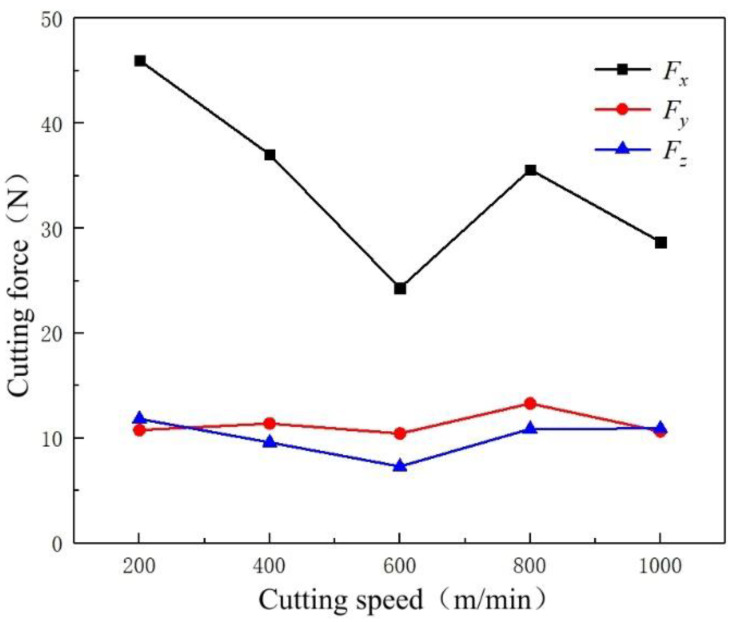
Variation trend of cutting force at different cutting speeds.

Figure 9 shows the variation trend of three-way cutting forces at different cutting speeds when milling T2 pure copper. The values of the cutting force *F_x_* are the highest in the tool-moving direction, and they significantly vary with an increase in the cutting speed. Cutting forces negatively correlate with the cutting speed, especially when the cutting speed is 600 m/min. Here, the value of *F_x_* is the lowest, but it slightly increases when the cutting speeds exceed 600 m/min. In the remaining two directions, the relationships between cutting forces and the cutting speeds do not significantly vary. Regarding numerical magnitude, *F_y_* and *F_z_* are close to each other and are much lower than *F_x_*. Based on the variation tendency perspective, the magnitude of *F_y_* is nearly constant at different cutting speeds. At the same time, *F_z_* and *F_x_* have a similar variation tendency, reaching the minimum value at 600 m/min.

Based on the principle of metal cutting, shear force *F_s_* can be expressed by the shear stress *τ* and the shear area *S* as follows:

(3) $$F_{s}=\tau \cdot S$$

and the shear area *S* can be expressed as follows:

(4) $$S=\frac{\mathrm{bh}}{\sin\phi }$$

where *b* is the cutting width, *h* is the actual cutting depth, and *ϕ* is the angle between the shear surface and the machining plane.

**Figure 10:**
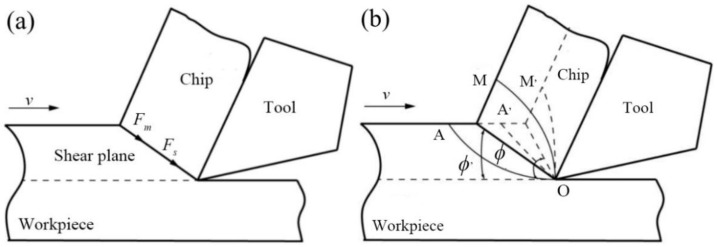
Effect of cutting speed on the shear deformation of workpieces: (**a**) shear plane stress; (**b**) shear angle variation.

The force required for the shear deformation of workpiece material during cutting mainly consists of the shear force *F_s_* and the chip inertia force *F_m_*, as shown in Figure 10a. Parameter *F_m_* can be expressed by Equation (5) [24]:

(5) $$F_{m}=\frac{{\rho \mathrm{bhv}}^{2}{\cos\alpha }_{r}}{\cos(\phi -\alpha_{r})}$$

where *ρ* is the machined material density, and *α_r_* is the tool rake angle. Parameter *F_m_* can be considered zero if the cutting speed is low due to the low chip mass.

Figure 10b illustrates the influence of cutting speed on the shear angle. With an increase in the cutting speed, the flow speed of workpiece material is higher than the plastic deformation speed, which would make the original shear deformation area AOM move back to the A’OM’ area as a whole, resulting in a shear angle *ϕ*’ > *ϕ*, as shown in Figure 10b. According to Equation (4), an increase in the shear angle leads to a decrease in shear force. Thus, the cutting force tends to decrease as the cutting speed increases. Meanwhile, the increase in the cutting speed will also increase the cutting temperature, decreasing the friction coefficient *μ* [24] and the thermal softening phenomena of the workpiece materials [25], further reducing the cutting force. When the cutting speed exceeds 600 m/min, it can be deduced from Equation (5) that the higher cutting speed may cause an increase in the inertia force *F_m_*, resulting in a slight increase in the cutting force. However, in terms of the overall trend, the cutting force still shows a decreasing trend.

#### 3.2.2. The Effect of Feed Rates

**Figure 11:**
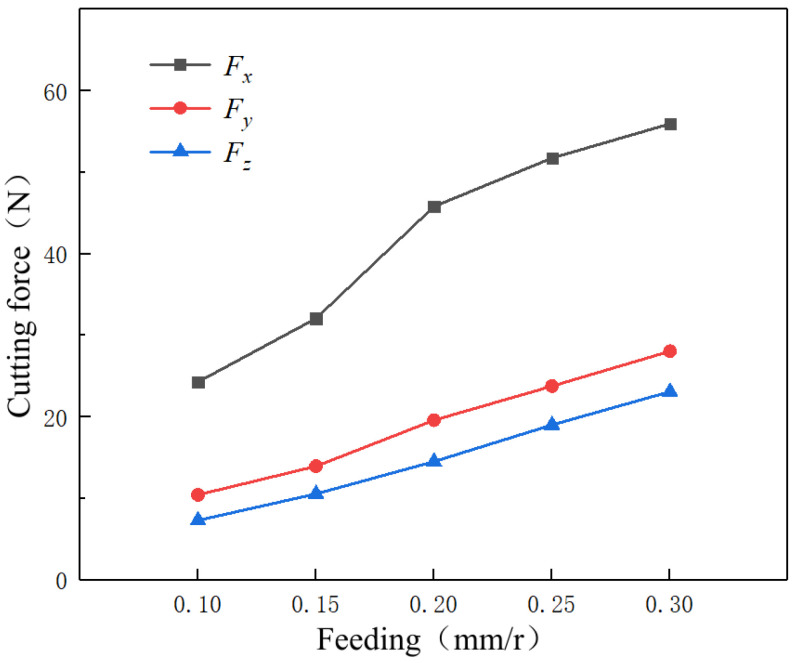
Variation trend of the cutting force at different feed rates.

Figure 11 represents the variation law of three-way cutting forces at different feed rates. The three-way cutting forces *F_x_*, *F_y_*, and *F_z_* show a positive correlation trend with an increase in the feed rate. This is because the increase in the feed rate will increase the cutting thickness in the feed direction of the tools, which results in additional workpiece material cut by the tools per unit of time, improving the reaction forces generated during plastic deformation of the workpieces. Finally, the three-way cutting forces are increased. Compared with the trend of the three-dot plots, an increase in cutting force is mainly concentrated in the tool moving direction *X*, which is also the predominant direction of the workpiece material deformation during milling and which is consistent with the actual machining conditions. Since material deformation and friction between the workpieces and the tools in the *Y* and *Z* directions are low, an increase in the cutting forces is relatively low.

#### 3.2.3. The Effect of Axial Cutting Depths

**Figure 12:**
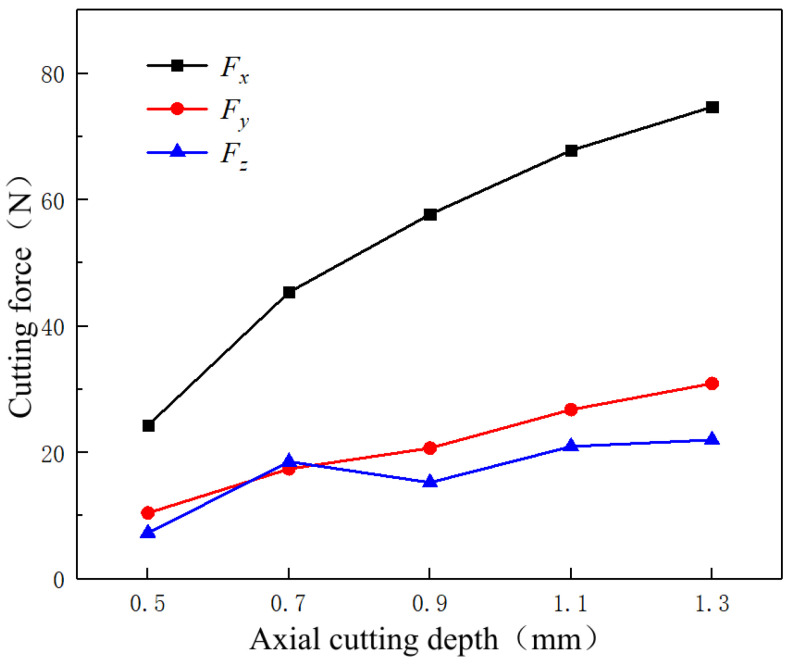
Variation trend of cutting force at different axial cutting depths.

Figure 12 shows the variation law of three-way cutting forces with different axial cutting depths in the milling process. With an increase in the axial cutting depth, the three-way cutting forces *F_x_*, *F_y_*, and *F_z_* show an upward trend. Similar to the factor of the feed rate, an increase in the cutting depth directly leads to an increase in the material volume of the cut workpiece per unit of time. Hence, the generated reaction forces during the plastic deformation of the materials are improved, and three-way cutting forces are increased.

In general, the cutting force shows a downward trend with increasing cutting speed, while increasing the feed rate and axial cutting depth can cause a sharp increase in the cutting force. Hence, an increase in the cutting force will affect the displacement acceleration states of the tools, increase the tool wear degree and damage the surface quality of the workpieces [26].

### 3.3. The Effect of Milling Processing Parameters on the Tool Displacement Acceleration and the Machined Surface Morphologies

#### 3.3.1. The Effect of Cutting Speeds

**Figure 13:**
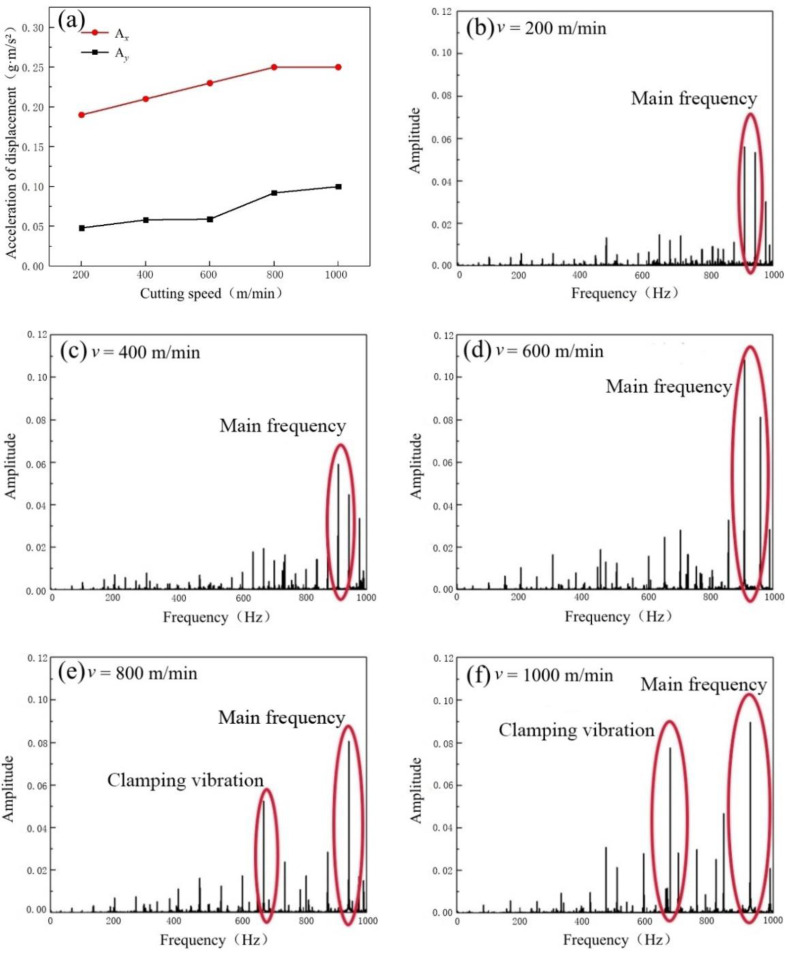
(**a**) Variation trends of displacement acceleration at different cutting speeds. The frequency domain distribution of tool-moving direction: (**b**) *v* = 200 m/min; (**c**) *v* = 400 m/min; (**d**) *v* = 600 m/min; (**e**) *v* = 800 m/min; (**f**) *v* = 1000 m/min.

Figure 13a illustrates the variation trends of displacement acceleration of the machine spindle in *X* and *Y* directions at different cutting speeds. In terms of numerical magnitude, the *X* acceleration in the tool-moving direction is much higher than that in the *Y* direction, similar to the cutting force case. However, the acceleration in both *X* and *Y* directions is increased with the cutting speed, which is exactly opposite to the variation trend of the cutting force. This could be explained as follows. When the milling speed is at a high level, with an increase in the feed rate, the tools need to cut into the workpieces with a higher frequency, causing the milling process to have a higher processing frequency. Moreover, the cutting force fluctuation frequency is higher than those at low-speed conditions, which aggravates the milling process instability, resulting in the machining tool vibration [27].

The frequency domain distributions of tool displacement acceleration under different cutting speeds are then obtained via the Fast Fourier Transform of the measured displacement acceleration signal in the tool-moving direction, as shown in Figure 13b–f. In the frequency range from 800 to 1000 Hz, there is the maximum amplitude of displacement acceleration, namely the natural frequency of the machine spindle. At low cutting speeds (200 and 400 m/min), the amplitudes are mainly concentrated at the main frequency position, indicating that the tool displacement acceleration mainly results from the forced vibration of the machine spindle, as shown in Figure 13b,c. However, at high cutting speeds (600, 800, and 1000 m/min), the amplitudes of the main frequency are significantly increased compared to those at the low-speed conditions, indicating that the process is in a relatively unstable condition under the influence of short-term and high-frequency cutting force fluctuation. In particular, at cutting speeds of 800 and 1000 m/min, the rapid rotation of the spindle causes severe vibration of the tool clamping structure, causing the middle spectrum positions to have a high amplitude, as shown in Figure 13e,f.

**Figure 14:**
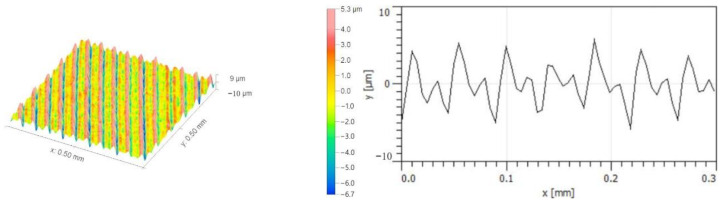
Micro-morphologies of machined surfaces with a cutting speed at *v* = 600 m/min.

Figure 14 illustrates the micro-morphologies of the machined surfaces with a cutting speed *v* = 600 m/min. In addition, the micro-morphologies of the machined surfaces with a low cutting speed (*v* = 200 m/min) and a high cutting speed (*v* = 1000 m/min) were reported in a previous work conducted by the authors [28]. Under the same milling feed rate and axial cutting depth, the residual height of the machined surfaces presents highly regular peak-valley morphologies. Comparison of the surface residual height profiles shows that the height difference between the peak valleys of the machined surfaces is lower at high cutting speed levels than those at low cutting speed levels. Hence, increasing the cutting speed may improve the surface quality of the workpieces. When the cutting speed is 600 m/min, the height difference of the workpiece surfaces is the lowest. The above-conducted analysis of tool displacement acceleration at different cutting speeds has shown that an increase in the cutting speed leads to the displacement acceleration of the machine spindle, triggering a deviation of the tool path during milling, superimposing a layer of periodic relative motions in the conventional milling path [29], and finally resulting in a variation of the surface morphologies.

#### 3.3.2. The Effect of Feed Rates

**Figure 15:**
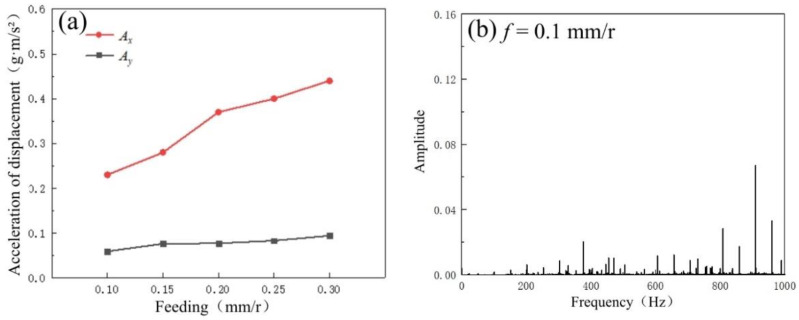

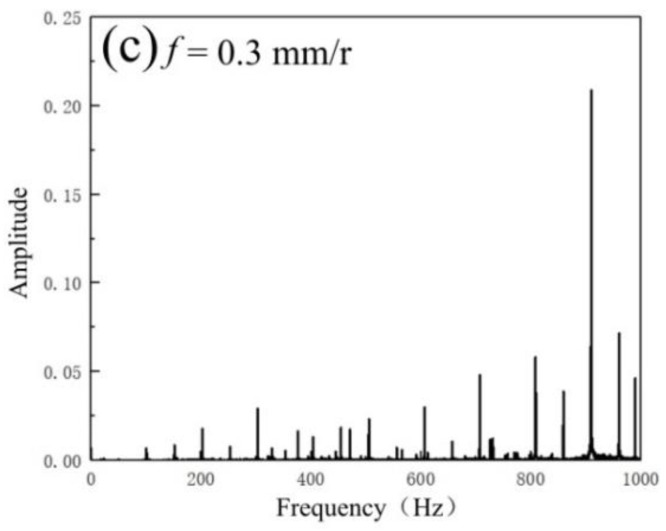
(**a**) Variation trends of displacement acceleration at different feed rates. The frequency domain distributions of tool-moving direction: (**b**) *f* = 0.1 mm/r; (**c**) *f* = 0.3 mm/r.

Figure 15a shows the variation trends of displacement acceleration of the machine spindle in the *X* and *Y* directions. The displacement acceleration increases with the feed rate. Moreover, the acceleration of *X* in the tool-moving direction is significantly increased, which is also consistent with the upward trend of the cutting force mentioned above, indicating that the increase in the cutting force may increase the displacement acceleration. To further confirm the source of the vibration, a frequency domain analysis is performed on the lowest and highest groups of feed rate in the milling tests, as shown in Figure 15b,c. When the feed rate increases, only the amplitudes are increased significantly in the main frequency band but not in the middle- and low-frequency bands. This is further evidence that an increase in the cutting force is the key factor causing an increase in displacement acceleration during milling when the feed rate is changed. However, other displacement acceleration sources, such as clamping vibration, have a relatively minor effect on milling [30].

**Figure 16:**
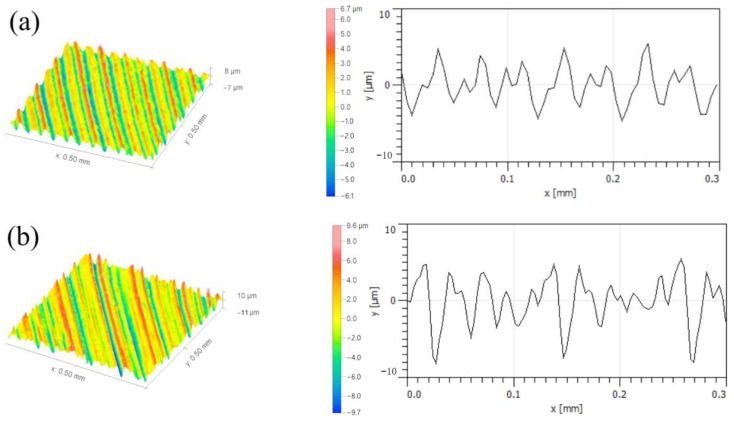
Micro-morphologies of machined surfaces at different feed rates: (**a**) *f* = 0.1 mm/r; (**b**) *f* = 0.2 mm/r.

Figure 16 shows the micro-morphologies of the machined surfaces at different feed rates. It can be seen that with an increase in the feed rate, the spacings of the profile periods of the machined surfaces are also gradually increased. Based on the influence of feed rate on the tool displacement acceleration conditions during the abovementioned milling, the height difference between the peak valleys in the surface profile periods is increased significantly due to an increase in the cutting force. Moreover, with the influence of the tool displacement acceleration deviation, the spacings between the profiles produce more dense tool marks, leading to a continuous increase in the surface roughness.

#### 3.3.3. The Effect of Axial Cutting Depths

**Figure 17:**
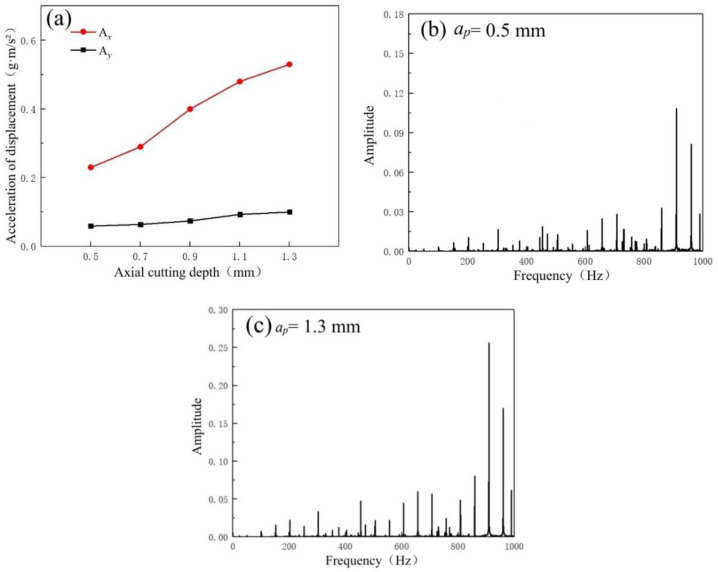
(**a**) Variation trends of displacement acceleration at different axial cutting depths. The frequency domain distribution of tool-moving direction: (**b**) *a_p_* = 0.5 mm; (**c**) *a_p_* = 1.3 mm.

According to Figure 17a–c, the variation trends of the displacement acceleration of the machine spindle in *X* and *Y* directions at different axial cutting depths and the frequency domain distributions of the tool moving direction are similar to the variation trends influenced by the feed rate. Moreover, the results conform to the abovementioned variation trends in the cutting force. The experimental results indicate that an increase in the cutting force caused by the change in the axial cutting depth is the predominant reason for an increase in tool displacement acceleration during milling.

**Figure 18:**
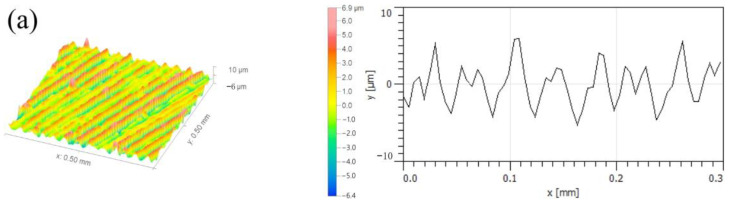

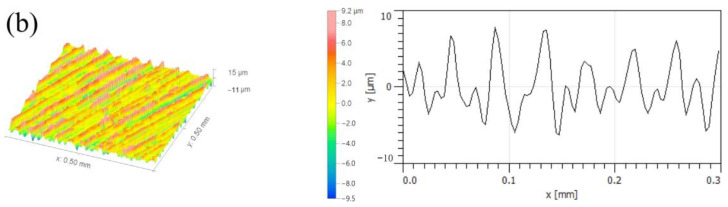
Micro-morphologies of machined surfaces at different axial cutting depths: (**a**) *a_p_* = 0.5 mm; (**b**) *a_p_* = 1.3 mm.

Figure 18 illustrates the micro-morphologies of the machined surfaces at different axial cutting depths. Since the cutting speed and feed rate remain constant, the spacings of the profile periods of the machined surfaces are consistent. According to the influence of the axial cutting depth on the tool displacement acceleration conditions during milling, the stability of the milling process is remarkably influenced by an increase in the cutting force and tool displacement acceleration. Hirose et al. [31] established a dynamic cutting layer model and concluded that the variation of cutting thickness will further aggravate the change of cutting force, which in turn affects the surface quality of the workpiece. Thus, when the axial cutting depth is at a high level, the height difference between the peak valleys of the machined surfaces is high, and the surface fluctuation is obvious. As a result, the surface roughness of the machined surfaces increases with the axial cutting depth.

## 4. Conclusions

In this paper, the effects of the T2 pure copper milling processing parameters on the tool cutting forces and tool displacement acceleration were studied by series milling experiments. Moreover, the surface quality of machined surfaces was evaluated and analyzed via surface roughness parameters. The conclusions can be drawn as follows:

(1) Based on the results of orthogonal milling tests of T2 pure copper, the cutting speed *v* had the highest degree of influence on surface roughness parameters of *S_a_* and *S_q_*, followed by the feed rate *f* and axial cutting depth *a_p_*. When *v* = 600 m/min, *a_p_*= 0.5 mm, *f* = 0.1 mm/r, *S_a_* and *S_q_* are 1.80 and 2.25 μm, respectively. The single-factor experiments showed that the values of *S_a_* and *S_q_* were relatively high, and the surface quality of the workpieces was poor at a low cutting speed. With an increase in the feed rate and axial cutting depth, *S_a_* and *S_q_* showed an upward trend.
(2) The cutting force *F_x_* in the tool-motion direction negatively correlate with the cutting speed. At a cutting speed of 600 m/min, *F_x_* reached the lowest value, and the value of *F_x_* was much higher than *F_y_* and *F_z_*. The three cutting forces showed a positive correlation with an increase in the feed rate and axial cutting depth. An increase in the cutting force was mainly observed in the *X* cutting direction, while the increase in the *Y* and *Z* directions was relatively low.
(3) The tool displacement acceleration amplitudes showed an upward trend with an increase in each milling processing parameter. The height difference between the peak valleys of the machined surfaces was lower when the cutting speed was at a high level than when the cutting speed was at a low level. When the feed rate was taken as a single factor, the spacing variation of the profile periods of the machined surfaces was more significant than when the axial cutting depth was taken as a single factor. It can be inferred that the cutting force and tool displacement acceleration seriously influenced the stability of milling machining.
(4) Combined with the orthogonal and single-factor test results, the optimized processing parameters for T2 pure copper milling were *v* = 600 m/min, *a_p_* = 0.5 mm, and *f* = 0.1 mm/r.

The variation in displacement acceleration states of the tools causes the machined surfaces to behave in different 3D morphologies. Furthermore, the wear degree of the tool is an important factor affecting the surface quality, which has been investigated and discussed in the previous research carried out by the authors [28]. In future work, internal relationships between the tool wear degree and the machined surface quality will be studied based on the tool displacement acceleration state.

## Figures and Tables

**Table 1:** Chemical compositions of T2 pure copper (wt.%).

Element	Ag + Cu	Bi	Sb	As	Fe	Pb	S
Content	99.90	0.001	0.002	0.002	0.005	0.005	0.005

**Table 2:** Cutting force and tool displacement acceleration test parameters for the T2 pure copper milling.

Independent Variables	Level of Parameters					Other Parameters
Cutting speeds *v* (m/min)	200	400	600	800	1000	*a_p_* = 0.5 mm, *f* = 0.1 mm/r, *a_e_* = 4 mm
Feed rates *f* (mm/r)	0.1	0.15	0.2	0.25	0.3	*v* = 600 m/min, *a_p_* = 0.5 mm, *a_e_* = 4 mm
Axial cutting depths *a_p_* (mm)	0.5	0.7	0.9	0.11	0.13	*v* = 600 m/min, *f* = 0.1 mm/r, *a_e_* = 4 mm

**Table 3:** Three-dimensional surface roughness parameters.

Symbols	Descriptions	Expressions	Meaning of Parameters
*S_a_*	Surface arithmetic mean deviation	$S_{a}=\frac{1}{A}\underset{A}{\iint }\left|\eta (x,y)\right|\mathrm{dxdy}$	*A* is the sampling area, and *η* (*x*, *y*) is the height difference between the sampling surface and the datum surface.
*S_q_*	Surface root mean square deviation	$S_{q}=\sqrt{\frac{1}{A}\underset{A}{\iint }\eta^{2}(x,y)\mathrm{dxdy}}$	

**Table 4:** Experimental designs and results of orthogonal milling.

Levels		Milling Parameters				Indexes	
		Cutting Speeds *v* (m/min)	Axial Cutting Depths *a_p_* (mm)	Feed Rates *f* (mm/r)	Blank Column	*S_a_* (*μm*)	*S_q_* (*μm*)
Test numbers	1	400	0.5	0.1	(1)	3.25	3.66
	2	400	0.7	0.15	(2)	3.76	4.42
	3	400	0.9	0.2	(3)	4.33	5.27
	4	600	0.5	0.15	(3)	2.21	2.8
	5	600	0.7	0.2	(1)	2.89	3.58
	6	600	0.9	0.1	(2)	2.59	3.21
	7	800	0.5	0.2	(2)	3.35	3.93
	8	800	0.7	0.1	(3)	2.88	3.47
	9	800	0.9	0.15	(1)	3.17	3.71

**Table 5:** *S_a_* range calculation of orthogonal test.

Main Effect Corresponding to the Indexes	Cutting Speeds *v* (a)	Axial Cutting Depths *a_p_* (b)	Feed Rates *f* (c)	Blank Column
*K*_1_	11.34	8.82	8.73	9.30
*K*_2_	7.68	9.54	9.15	9.69
*K*_3_	9.39	10.08	10.56	9.42
Range (*R*)	3.66	1.26	1.83	0.39
Primary and secondary factors order	*v* > *f* > *a_p_*			

**Table 6:** *S_q_* range calculation of orthogonal test.

Main Effect Corresponding to the Indexes	Cutting Speeds *v* (a)	Axial Cutting Depths *a_p_* (b)	Feed Rates *f* (c)	Blank Column
*K*_1_	13.35	10.38	10.35	10.95
*K*_2_	9.60	11.46	10.92	11.56
*K*_3_	11.10	12.18	12.78	11.54
Range (*R*)	3.75	1.80	2.43	0.61
Primary and secondary factors order	*v* > *f* > *a_p_*			

## Data Availability

The data presented in this study are available in article.
